# Parental Awareness and Practices Regarding Electronic Devices’ Effects on Children's Sleep: A Cross‐Sectional Study

**DOI:** 10.1002/puh2.70300

**Published:** 2026-06-15

**Authors:** Ahoud Ali Al‐Shammary, Nouf Fahad Aldawsari, Rana Ali Alharfi, Alhanouf Abdullah Ibrahim Almujil, Yousef Abud Alanazi, Mohammad Shakil Ahmad, Hamza Abdulmunem Orfali

**Affiliations:** ^1^ College of Medicine Majmaah University Majmaah Saudi Arabia; ^2^ Department of Pediatrics College of Medicine Majmaah University Majmaah Saudi Arabia; ^3^ Department of Family & Community Medicine College of Medicine Majmaah University Al‐Majmaah Saudi Arabia; ^4^ Obstetrics and Gynecology Department Al Neelain University Khartoum Sudan

**Keywords:** parental awareness, sleep patterns, smartphones

## Abstract

**Background:**

Adequate sleep is essential for children and adolescents, supporting mental, physical, and academic development. Increasingly, widespread use of digital devices, including smartphones, tablets, and video games, has been linked to poor sleep quality, delayed bedtimes, and behavioral issues. Excessive screen time, particularly before bedtime, can disrupt sleep patterns and increase the risk of emotional and cognitive problems. Understanding parental awareness, beliefs, and practices regarding children's use of smart devices is crucial to mitigate these negative effects.

**Methods:**

A cross‐sectional study was conducted from November 2023 to May 2024 among 400 parents of healthy children and adolescents in Majmaah. Data were collected through online‐based questionnaires assessing on the basis of educational level, complete structured questionnaire covering demographics, beliefs, and practices. Analysis was performed using SPSS Version 27, applying chi‐square and *t*‐tests with a significance level of *p* < 0.05.

**Results:**

The study included 400 parents, predominantly females (79.75%) and married (66.5%), with most holding a bachelor's degree (49%) and having more than four children (41.5%). Most parents (86.75%) were aware of the negative effects of smart devices, particularly on sleep (93%) and vision (92.75%). Although 77.5% enforced device limits and 86% sought medical care for related symptoms, many children still exceeded recommending screen time, affecting sleep and behavior. Parental education was significantly associated with awareness (*p* < 0.05). Findings highlight that despite high awareness, consistent regulation of device use remains challenging, especially in younger children.

**Conclusion:**

This study assessed parents’ awareness and practices regarding children's smart device use in Majmaah. Educated parents recognized risks to sleep and vision, yet many struggled to limit usage, especially in children aged 0–5 years. Parental education correlated with awareness, and device use affected sleep. Findings highlight the need for parental guidance and structured screen time limits to protect children's health.

## Graphical abstract

This graphical abstract summarizes a cross‐sectional study of 400 parents in Majmaah, Saudi Arabia. Despite high awareness of the negative effects of electronic devices on sleep, consistent regulation of screen time remains challenging, especially among younger children. Parental education was significantly associated with higher awareness.

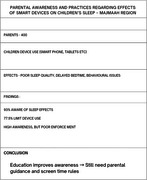

## 1 Introduction

Adequate sleep is critical for children and adolescents, supporting mental and physical health. Infants require 16–18 h per night, decreasing to around 10 h during childhood, whereas adolescents often fail to meet these needs [1]. Insufficient sleep is linked to emotional disturbances, obesity, metabolic risk, and poor academic performance [1]. Increasingly, digital media, including smartphones, tablets, computers, and video games, affects sleep patterns in young populations [2, 3]. Video game addiction can worsen mental health issues, including anxiety, depression, and inattention, with gaming disorders affecting 1%–15% of teenagers globally. In Saudi Arabia, 11.4% of adolescents experience high stress, and non‐Saudi adolescents may be more vulnerable to addiction [4].

Screen‐based activities before bedtime can disrupt sleep by altering neurotransmitters, delaying sleep onset, and reducing overall sleep quality [5]. The widespread integration of digital media into children's daily lives has raised parental concerns regarding potential harm [3]. Research indicates that frequent device use is associated with shorter sleep duration, delayed bedtimes, poor sleep quality, and related academic or behavioral problems [2, 6].

Large‐scale studies support these associations. Norwegian adolescents aged 16–19 who used multiple devices at bedtime had shorter sleep duration and longer sleep onset latency, showing a dose response relation [5]. In Italy, 63.5% of children aged 1–14 used video devices before bed, with one‐third sleeping fewer hours than recommended [1]. Parental reports of infants’ media use also correlated with delayed sleep and reduced duration [3]. Slovak adolescents screen time was linked to poor sleep quality, which mediated academic difficulties [6]. In Saudi Arabia, adolescents using screens frequently, especially before bedtime, showed poor sleep quality, daytime sleepiness, and fatigue [7]. Another local study found that tablets and smartphones use from more than 3 to 5 h negatively impacted children's sleep and behavior [8]. Across 25 studies, 90% reported negative associations between screen‐time and sleep outcomes, varying by device type and usage patterns.

This study aims to examine the relationship between smart device use and children's sleep patterns, focusing on parental awareness, beliefs, and practices. Understanding these factors is crucial to reduce sleep disturbances and promote healthy development in children and adolescents.

## 2 Methodology

This cross‐sectional descriptive study was conducted in Al Majmaah City, kingdom of Saudi Arabia, to assess parents’ awareness and practices regarding the effects of electronic devices on children's health. The study targeted parents of children and adolescents under 16 years of age attending local schools. The sample size was calculated using the single proportion formula *n* = *z*2*p*(1 − *p*)/*d*2, with a 95% confidence level (*z* = 1.96), *p* = 0.52, and a 5% margin of error, resulting in 383 participants; to compensate for potential non‐response, the final sample size was set at 400. A stratified random sample sampling method was applied to ensure proportional representation of parents from different educational levels (illiterate, secondary, diploma, bachelor, and postgraduate). Data were collected over 5 months using a self‐administered structured questionnaire distributed electronically and in printed form after obtaining informed consent. The questionnaire included three sections: the first collected demographic information (such as gender, age, martial status, education, and number of children); the second assessed parental beliefs toward smart devices (six items evaluating awareness of risks, appropriate usage age, and perceived health effects); and the third examined parental practices (seven items on screen‐time management and health‐seeking behavior). Items were close‐ended and formatted as multiple choice or Likert‐scale questions. Data were analyzed using SPSS version 27. Descriptive statistics and chi‐square test were used a *p* value of less than 0.05 was considered statistically significant. Ethical approval was obtained from institutional review committee, and participants were voluntary. Privacy and confidentiality were maintained by anonymizing responses, and data were used exclusively for research purposes. Inclusion criteria compromised Saudi and non‐Saudi male and female parents residing in Al Majmaah with at least one child under 16 years, whereas adults without children and those living outside the region were excluded.

## 3 Results

Table 1 shows that the majority of individuals for a sample are “female” by 79.75%, and it was found that the marital status variable was the most of the sample members of the “married” category by 66.50%, and 33.50% were either divorce or widows, as for educational level where most of the participants were from the category of “bachelor” with 49.00% and the lowest category “illiterate” by 8%, and the number of children among the participants was the most percentage group “more than four,” 41.50% and the lowest category “1” by 10.25%, and the age of children, where most of the category was “0–5 years” by 45.00%, followed by the category “6–10 years” by 41.50%, followed by category “11–15 years” by 13.50%.

**TABLE 1:** Sociodemographic status of the parents of children less than 16 years old.

Variable	Number	Percentage
**Gender**		
Female	319	79.75
Male	81	20.25
**Marital status**		
Married	266	66.50
Divorced	116	29.00
Widow	18	4.50
**Educational level**		
Bachelor	196	49.00
Secondary	81	20.25
Diploma	54	13.50
Post‐graduation	37	9.25
Illiterate	32	8.00
**Number of children**		
1	41	10.25
2	56	14.00
3	67	16.75
4	70	17.50
More than 4	166	41.50
**Age (years) of the youngest child**		
0–5	180	45.00
6–10	166	41.50
11–15	54	13.50

Table 2 shows the extent to which parents know about the harms of smart devices on sleep in children. For the study sample, it was found that in question No. 1, 13.25% (53) of the parents disagree with this statement, and 86.75% (347) agree with it. Then, followed by question No. 2, the number of parents who agreed with this statement was 19.25% (77), and the number of parents who did not agree with this statement was 80.75% (323). In addition, in question No. 3, 93.0% (372) of parents agree with this statement, and 7% (28) of parents disagree with it. In addition, question number 4, in which the number of parents who agree with this statement is 92.75% (371) and the number of parents who do not agree with this statement is 7.25% (29). Followed by question No. 5, the number of parents who agreed with this statement was 82.25% (329), and the number of parents who did not agree with this statement was 17.75% (71). In question No. 6, 55.75% (223) of parents agree with this statement, and 44.25% (177) of parents disagree with it.

**TABLE 2:** General awareness of parents toward the effect of smart devices on children's health.

General awareness of parents	No	Yes
Have you heard or read about the impact of smart devices on children's health?	(53) 13.25%	(347) 86.75%
If the device does not cause any symptoms, there is no issue with giving it to him all the time	(323) 80.75%	(77) 19.25%
Extended periods of sitting and playing video games can potentially cause sleep problems in children	(28) 7.00%	(372) 93.00%
Smart devices may contribute to poor vision and visual problems	(29) 7.25%	(371) 92.75%
Children should not possess a smart device until the age of 12	(71) 17.75%	(329) 82.25%
Can both playing for long continuous hours and not wearing lenses for protection from blue lights put children at risk?	(177) 44.25%	(223) 55.75%

Table 3 shows the practices and behaviors of children in using the smart devices/tablets on the basis of the answers of parents’, and in question No. 1 it shows that most of the parents sometimes set specific time for devices use by 53.5%, followed by question No. 2, it shows that 50.0% of the parents let their children use the devices unresponsively. Followed by question No. 3 which indicates that 66.75% of children use their devices for studying, lastly question No. 4 which shows that 65.75% of schools of the children focus on e‐learning.

**TABLE 3:** Parents’ practice toward children's use of smart devices.

Practice	Rarely	Sometimes	Always
Do you set a specific time to use smart devices?	(42) 10.5%	(214) 53.5%	(144) 36.0%
Do you let your child use their smart devices unsupervised?	(136) 34.0%	(200) 50.0%	(64) 16.0%
Do your children always use smart devices to study?	(47) 11.75%	(267) 66.75%	(86) 21.5%
Does the school focus on e‐learning?	(52) 13.0%	(263) 65.75%	(85) 21.25%

The table presents data on two key parental practices related to their children's use of smart devices and subsequent health issues. First, it shows that a significant majority of parents (77.5%) enforce strict rules when their children resist stopping smart device use, with 310 parents adhering to this approach compared to 90 parents (22.5%) who do not. Second, the data reveal that an even larger proportion of parents (86%) are proactive in seeking medical attention for symptoms related to smart device use, such as vision or sleep issues, with 344 parents taking this step versus 56 parents (14%) who do not. Overall, the results indicate that most parents take active steps to manage their children's smart device usage and address related health concerns, with a notable emphasis on seeking medical help when symptoms arise.

Table 4 shows that all *p* values are less than 0.05, indicating a significant association between educational level and awareness for each question in the data provided. Parents with a bachelor's degree consistently reported higher awareness across all indicators.

**TABLE 4:** Parental practices in managing children's smart device usage and addressing health symptoms.

Practice	Yes	No
Following a strict procedure when the child does not want to leave the smart devices	(310) 77.5%	(90) 22.5%
Going to the hospital if symptoms, such as poor vision or sleep problems appeared	(344) 86.0%	(56) 14.0%

## 4 Discussion

**FIGURE 1:**
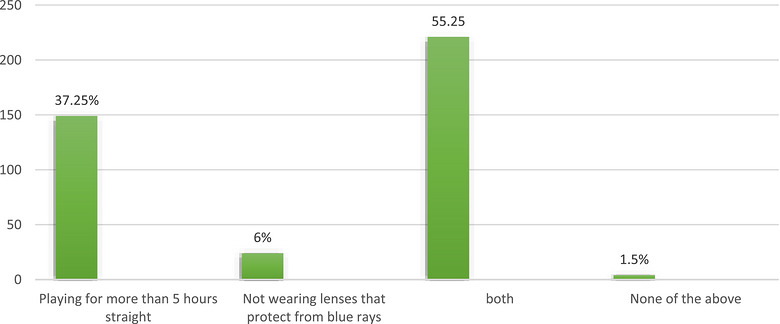
Awareness of parents of children less than 16 years old about the risks associated with smart devices.

In this study, we analyzed the characteristics of parents who responded on behalf of their children. Most respondents were female (79.75%), as shown in Table 1, consistent with studies emphasizing the influential role of mothers in children's smartphone use. Research indicates that mothers addicted to smart devices are a key factor in children's overuse [9], supporting our finding of mothers’ pivotal role. Regarding education, most participants held a bachelor's degree (49.00%), whereas only 8% were illiterate (Table 1). This suggests that higher parental education is linked to greater awareness of smartphone effects on children's sleep and stricter screen‐time regulation (Table 4, Figure 1). Conversely, less‐educated parents may have limited understanding and weaker control over device use. Previous studies have shown that children of less‐educated parents experience shorter sleep durations [10], aligning with our results that parental education influences sleep patterns (Table 4, Figure 1), with screen time being a major factor (Table 5).

**TABLE 5:** Impact of smart device use on children's behavior and sleep patterns.

Practice	Always	Sometimes	Rarely
Children miss their sleep schedule due to their use of smart devices	(164) 41%	(136) 34%	(100) 25%
Children avoid sleeping until the next day as a result of his use of smart devices	(93) 23.25%	(118) 29.5%	(189) 47. 25%
Children always use devices for more than 6 h daily	(57) 14.25%	(144) 36%	(199) 49.75%
A reduction in the number of hours children sleep due to their use of smart devices	(86) 21.5%	(159) 39. 75%	(155) 38.75%
Have you noticed changes in your child's behavior?	(262) 65.5%	(138) 34.5%	(0) 0%

The study showed that the largest group of parents had more than four children (41.50%) (Table 1), suggesting that larger families may be more aware of smartphone‐related health impacts yet face greater challenges in managing screen time. This aligns with previous studies showing that parents recognize the harmful effects of excessive smartphone use but struggle to regulate usage due to issues of autonomy and children's rights [11]. Moreover, 92.75% of parents agreed that “Smart devices may contribute to poor vision and visual problems” (Table 2), reflecting strong awareness of visual health risks. Prior research linked prolonged gadget use to decreased visual acuity and myopia [12], consistent with our findings that frequent smartphone use contributes to visual problems (Table 5), emphasizing the importance of monitoring and limiting screen time.

A large proportion of parents (82.25%) agreed that “Children should not have smart devices until age 12” (Table 4, Figure 1), showing strong support for limiting early exposure to enhance sleep quality. Previous studies found that nighttime screen use is linked to poor sleep outcomes, and lower health‐related quality of life in adolescents [13, 14], supporting our results. Additionally, 55.75% of parents acknowledged that prolonged play time exceeding 5 h and not wearing protective lenses poses risks to children (Table 2). These findings highlight the combined dangers of extended screen time and blue light exposure, both of which can worsen sleep problems. Studies confirm that blue light use at night reduces sleepiness and increases confusion [15]. This insight is critical for parents, educators, and healthcare providers, showing the need for comprehensive strategies to mitigate these risks and encouraging the use of blue light filters. And that the high screen time causes restless sleep and related symptoms [14]. Overall, our findings align with existing research indicating that prolonged smartphone use and blue light exposure negatively affect children's sleep.

**FIGURE 2:**
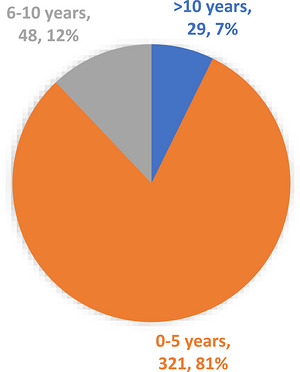
Parental awareness regarding the age at which children are most susceptible to the risks associated with smart devices.

Parents reported that children aged 0–5 years are the most vulnerable to the risks of smart device use (81%), followed by those aged 6–10 years (12%) and over 10 years (7%) (Figure 2). This indicates that younger children are particularly susceptible to the negative effects of smart devices. Recognizing this vulnerability can help parents and educators develop targeted strategies to manage and monitor screen time, reducing sleep disturbances and related issues. Previous study supports this finding, showing that higher evening and daily tablet or smartphone use among preschoolers aged 3–5 years is linked to sleep disturbances [16].

This part of the study explored parents’ behavior toward their children's use of smart devices/tablets and its impact on health and behavior. Parent's responses, shown as arithmetic means and standard deviations, indicate significant concern. For example, the statement “Would you go to the hospital if symptoms such as poor vision or sleep problems appeared?” had the highest mean of 1.91 (SD = 0.289) (Table 6), reflecting awareness of potential health risks. Previous studies support this, linking excessive smart device use to myopia [17], Computer Vision Syndrome (CVS) [18], and other health issues such as headaches, sleep disturbances, neck/shoulder pain, and transient vision loss [19]. The statement “Will you follow a strict procedure when the child does not want to leave the smart devices?” had a mean of 1.83 (SD = 0.374) (Table 6), indicating that many parents are inclined to implement strict rules to manage their children's device usage. Additionally, the statement “Have you noticed changes in your child's behavior?” had a mean of (1.69) (SD = 0.465) (Table 6), showing a notable observation among parents regarding behavioral changes and potentially linked to device use. Supporting this, a study in China found that low levels of physical activity and high sedentary behavior predict increased smartphone use, which is associated with poor sleep quality [20].

**TABLE 6:** The relation between parental awareness of smart device effects and their level of education.

Educational level	Heard about impact	No. issue with using the device	Sleep problems	Vision problems	No. device until 12	Risk from long play hours
Bachelor	168	6	173	184	190	100
Secondary	75	15	82	92	58	62
Diploma	60	35	68	63	32	40
Post graduation	30	87	32	18	27	15
Illiterate	14	180	17	14	22	6
**Total**	347	323	372	371	329	223
Chi‐square	**45.77**	**30.15**	**50.22**	**48.75**	**20.38**	**40.15**
*p* value	0.000001	0.0000046	0.000001	0.000001	0.00042	0.000001
Significant association	yes	yes	yes	Yes	yes	Yes

Results show a statistically significant positive relationship between smartphone use and children's sleep patterns (Table 5), with a Pearson correlation of 0.162 and Sig. 2‐tailed = 0.001, indicating that increased smartphone use alters sleep patterns. Previous studies confirm that high smartphone use is consistently linked to poor sleep quality, and smartphone addiction affects sleep independent of usage duration [21, 22].

A weak positive relationship was found between video game usage and children's health (*R* = 0.279, *R* ^2^ = 7.8%). The regression model was significant (*F *= 33.396, Sig. = 0.000), demonstrating that higher video game use slightly increases the health score. This indicates a weak link between smart devices and general health. Other studies suggest that short‐term interactive video games combined with traditional rehabilitation can improve physical health in children with developmental delays [23], and high video game use is linked to fewer peer relationship problems and prosocial deficits [24].

This study therefore builds on the existing evidence that smart devices can change children's behavior. Moreover, it shows that 65.5% of parents are noticing changes in their children's behaviors (Table 5). A systematic review found that 90% of studies reported a significant adverse association with at least one of the measured sleep outcomes that causes a change in children's sleep–wake behavior [2]. Most parents set a specific device usage time for their children: 36.0% set limits, whereas 10.5% do not (Table 3). Pathological internet use has been linked to shorter sleep insomnia, and weekend fatigue, possibly due to blue light suppressing melatonin [25]. Regarding e‐learning, 13% of schools rarely focus on it, whereas 21.25% emphasize it (Table 3). Students from high‐income schools tend to use more gadgets and may own personal cell phones compared to those in low‐income schools [26].

Regarding smart device use for studying, 21.5% of parents reported that their children always use devices for study, whereas 11.75% reported rarely use (Table 3). However, a recent study evaluates the quality of e‐learning used for their children since the coronavirus pandemic, showed that the parents were satisfied about the outcomes of using smart devices for learning [17].

## 5 Limitations of the Study

The study had several limitations. The sample size and participant selection may have introduced bias, limiting how well the results represent other populations. Some responses might not be fully accurate due to self‐reporting and social desirability bias. Time and resource constraints also restricted the depth of data collection and analysis. In addition, the findings may not fully apply outside the Majmaah context.

## 6 Conclusion

This study examined parents’ awareness, attitudes, and practice regarding children's use of smart devices in Majmaah. The findings revealed that most respondents were educated mothers, highlighting their key role in managing children's screen time. Parents showed high awareness of the negative health effects of excessive smart device use, particularly its impact on sleep and vision. However, despite this awareness, many reported challenges in consistently limiting usage. The results demonstrated significant association between parental education and awareness, as well as between smartphone use and altered sleep patterns among children. Younger children, especially those aged 0–5 years, were found to be most vulnerable to these risks. Although most parents implemented rules and sought medical help when symptoms appeared, prolonged screen exposure and limited physical activity remained common. Overall, the study underscores the need for greater parental guidance, structured screen‐time limits, and health education to mitigate the adverse effects of smart device use on children's well‐being.

## 7 Recommendations

1. Limit smartphone use among children, particularly during the evening and before bedtime.
2. Promote sleep hygiene education for both parents and children, emphasizing consistent bedtime routines and electronic‐free sleeping environment.
3. Increased awareness of the effects of blue light exposure on sleep quality, especially in younger children.
4. Encourage regular physical activity to counteract sedentary behaviors associated with smartphone use.
5. Support further research on the long‐term impacts of smart device use and the influence of different content types on children's health and environment.

## Author Contributions

**Ahoud Ali Al‐Shammary**: conceptualization, methodology, software, data curation, investigation, validation, formal analysis, supervision, visualization, project administration, writing – original draft, writing – review and editing. **Mohammad Shakil Ahmad**: supervision, resources, writing – original draft, writing – review and editing. **Rana Ali Alharfi**: methodology, software, data curation, resources, writing – original draft, writing – review and editing. **Hamza Abdulmunem Orfali**: writing – review and editing, conceptualization, validation, supervision. **Yousef Abud Alanazi**: supervision, writing – original draft, writing – review and editing. **Alhanouf Abdullah Ibrahim Almujil**: software, data curation, validation, investigation, writing – original draft, writing – review and editing. **Nouf Fahad Aldawsari**: methodology, data curation, investigation, formal analysis, visualization, writing – original draft, writing – review and editing.

## Funding

The authors have nothing to report.

## Conflicts of Interest

The authors declare no conflicts of interest.

## Data Availability

Data supporting the findings of this study are available from the corresponding author.
